# The complete chloroplast genome sequence of *Fragaria orientalis* (Rosales: Rosaceae)

**DOI:** 10.1080/23802359.2018.1424578

**Published:** 2018-01-12

**Authors:** Yan Han, Hai Bo Wu, Yuan Liu

**Affiliations:** Faculty of Life Science and Technology, Kunming University of Science and Technology, Kunming, China

**Keywords:** *Fragaria orientalis*, chloroplast genome, phylogenetic analysis

## Abstract

*Fragaria orientalis* (Rosales: Rosaceae) as drought-resisting plant is distributed throughout northeast and central Asia. The complete chloroplast genome sequence of *Fragaria orientalis* is reported and characterized in this study. The genome size of *F. orientalis* is 147,835 bp, containing a pair of inverted repeats (IR) regions of 51,216 bp, which were separated by a large single copy (LSC) region of 83,232 bp and a small single copy (SSC) region of 13,387 bp. The genome contains 128 genes, including 84 protein-coding genes, 36 transfer RNA (tRNA) genes, and eight ribosomal RNA (rRNA) genes. A total of 17 of these genes are duplicated in the IR regions, 16 genes contain one intron, and *ycf3* has two introns. Maximum likelihood phylogenetic analysis based on chloroplast genome sequences indicates that *F. orientalis* is closely related to *Fragaria chiloensis* and *Fragaria virginiana* from the family Rosaceae.

*Fragaria orientalis* were found in 1934, tetraploid, had 28 chromosomes (Fedorova 1934). *F. orientalis*, belonging to the Rosaceae family, is native to northeast and central Asia (Guan et al. 2004). In order to adapt the environment, *F. orientalis* has evolved the features that are resistant to cold and drought (Guo et al. 2015). *F. orientalis* is a good wild germplasm resource of strawberry breeding (Gu et al. 2010). As a member of strawberry, *F. orientalis* is usually propagated by the stolon, so the fruit is small, and yield is low (Gu et al. 2010; Sun et al. 2010). *F. orientalis* tastes like bubblegum which is popular with Europe (http://www.dailymail.co.uk/sciencetech/article-2616658).

Based on the widely distribution, *F. orientalis* may have more genetic diversity than the species with narrow distribution. We will find the new taste strawberry by developing the genetic diversity of *F. orientalis*. So, our study had got the completion of chloroplast genome of *F. orientalis*, it will not only enhance the conservation efforts of this plant species, but also provide the plentiful gene resources for *Fragaria* breeding.

The materials of *F. orientalis* come from the NCGR-Corvallis (National Center for Genome Resources); the sequence platform is Illumina HiSeq1000, and the total bases are about 106 GB (Hirakawa et al. 2014). In the study, we use the raw data of *F. orientalis* (Hirakawa et al. 2014) to get the complete chloroplast genome sequence (GenBank: KY769126). We used the CLC Genomics Workbench v. 3.6.1 (CLC Inc., Aarhus, Denmark) to filter and assemble contigs. The circular map was obtained using the online program OGDRAW (Lohse et al. 2013).

The *F. orientalis* chloroplast genome is 147,835 bp long, with a typical circular structure, containing a pair of inverted repeat (IR) regions of 25,608 bp, which were separated by a large single copy (LSC) region of 83,232 bp and a small single copy (SSC) region of 13,387 bp. The chloroplast genome contains 128 genes, including 84 protein-coding genes, 36 transfer RNA (tRNA) genes, and eight ribosomal RNA (rRNA) genes. Most of these genes occur as a single copy, however, seven protein-coding genes, seven tRNA genes, and four rRNA genes are duplicated in the IR regions. A total of 17 of these genes are duplicated in the IR regions, 16 genes contain one intron, especially, the gene *ycf3* has two introns. The overall GC content of the chloroplast genome is 37.60% and the corresponding values in LSC, SSC, and IR regions are 35.23%, 30.22%, and 42.86%, respectively.

We constructed a maximum likelihood phylogenetic tree using seven species from the order Rosales (Figure 1), while *Glycine soja* and *Triticum aestivum* served as outgroup. All the chloroplast genome sequences were aligned using MEGA6 (Kumar et al. 2016). Our result showed that *F. orientalis* is most closely related to *F. chiloensis* and *F. virginiana* from the family Rosales. The complete chloroplast genome of *F. orientalis* would provide valuable information for biodiversity conservation and genetic breeding.

**Figure 1.**
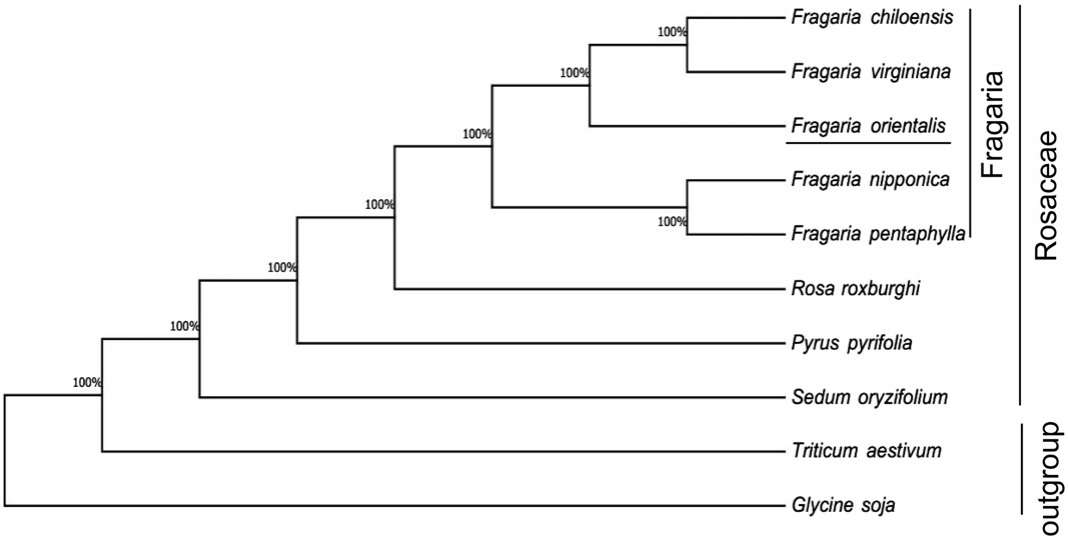
Maximum likelihood phylogenetic tree of *F. orientalis* with seven other species in the Rosales based on complete chloroplast genome sequences, while using *Glycine soja* and *Triticum aestivum*as outgroup. Accession numbers are listed as below: *Fragaria chiloensis* (NC_019601), *Fragaria nipponica* (NC_035500), *Fragaria pentaphylla* (NC_034347), *Fragaria virginiana* (NC_019602), *Pyrus pyrifolia* (NC_015996), *Rosa roxburghii* (NC_032038), *Sedum oryzifolium* (NC_027837), *Glycine soja* (NC_022868.1), and *Triticum aestivum* (NC_002762).
